# Improvement of cardiac contractile function by peptide-based inhibition of NF-κB in the utrophin/dystrophin-deficient murine model of muscular dystrophy

**DOI:** 10.1186/1479-5876-9-68

**Published:** 2011-05-17

**Authors:** Dawn A Delfín, Ying Xu, Jennifer M Peterson, Denis C Guttridge, Jill A Rafael-Fortney, Paul ML Janssen

**Affiliations:** 1 Department of Molecular and Cellular Biochemistry, Columbus, OH, USA; 2 Department of Physiology and Cell Biology, Columbus, OH, USA; 3 Department of Molecular Virology, Immunology, and Medical Genetics, The Ohio State University, Columbus, OH, USA

## Abstract

**Background:**

Duchenne muscular dystrophy (DMD) is an inherited and progressive disease causing striated muscle deterioration. Patients in their twenties generally die from either respiratory or cardiac failure. In order to improve the lifespan and quality of life of DMD patients, it is important to prevent or reverse the progressive loss of contractile function of the heart. Recent studies by our labs have shown that the peptide NBD (Nemo Binding Domain), targeted at blunting Nuclear Factor κB (NF-κB) signaling, reduces inflammation, enhances myofiber regeneration, and improves contractile deficits in the diaphragm in dystrophin-deficient *mdx *mice.

**Methods:**

To assess whether cardiac function in addition to diaphragm function can be improved, we investigated physiological and histological parameters of cardiac muscle in mice deficient for both dystrophin and its homolog utrophin (double knockout = dko) mice treated with NBD peptide. These dko mice show classic pathophysiological hallmarks of heart failure, including myocyte degeneration, an impaired force-frequency response and a severely blunted β-adrenergic response. Cardiac contractile function at baseline and frequencies and pre-loads throughout the in vivo range as well as β-adrenergic reserve was measured in isolated cardiac muscle preparations. In addition, we studied histopathological and inflammatory markers in these mice.

**Results:**

At baseline conditions, active force development in cardiac muscles from NBD treated dko mice was more than double that of vehicle-treated dko mice. NBD treatment also significantly improved frequency-dependent behavior of the muscles. The increase in force in NBD-treated dko muscles to β-adrenergic stimulation was robustly restored compared to vehicle-treated mice. However, histological features, including collagen content and inflammatory markers were not significantly different between NBD-treated and vehicle-treated dko mice.

**Conclusions:**

We conclude that NBD can significantly improve cardiac contractile dysfunction in the dko mouse model of DMD and may thus provide a novel therapeutic treatment for heart failure.

## Background

Duchenne muscular dystrophy (DMD) is a degenerating striated muscle disease caused by the absence of the dystrophin protein[1]. Although limb muscle weakness and the loss of ambulation are usually the initial clinical signs of the disease, patients with DMD die from respiratory failure or heart failure. Pertaining to the heart, ninety-five percent of DMD patients develop dilated cardiomyopathy, and over twenty-five percent die from heart failure [2]. These numbers are predicted to grow as prophylactic treatments targeted at maintaining respiratory function improve[3]. This prediction is further supported by the majority of patients with Becker muscular dystrophy (BMD), who have dystrophin mutations that cause a milder skeletal muscle disease, and typically progress to heart failure[3].

Improving skeletal muscle function has been the central focus of therapeutic development for DMD and BMD. However, therapies targeting only skeletal muscle but not cardiac muscle could potentially aggravate the already present cardiac dysfunction[4]. In order to improve lifespan and quality of life, progressive loss of contractile function in the heart also needs to be prevented or halted. Our recent studies have shown that the inhibition of the NF-κB signaling pathway can improve both limb and diaphragm muscle contractile function in the dystrophin-deficient *mdx *genotypic mouse model of DMD[5,6]. This inhibition was achieved by a small, 11 amino-acid peptide named NBD (NEMO Binding Domain) that binds preferentially to the C-terminal regions of the IKKα and IKKβ catalytic components of IκB kinase (IKK) preventing association with the NF-κB essential modulator (NEMO) regulatory subunit and prohibiting downstream NF-κB signaling. The NBD peptide blunted NF-κB signaling, reduced inflammation, enhanced myofiber regeneration, and improved contractile function in the diaphragm muscle in *mdx *mice[5,6].

It is interesting to note that of the pharmacological inhibitors tested for improvement of skeletal muscles in animal models of DMD, none, to our knowledge, were directly tested for their effects to improve cardiac function. Recent studies even suggest that the current standard of care pharmacological treatment for DMD, the corticosteroid prednisone, worsens cardiac function in the *mdx *model[7,8]. It is not known whether cardiac contractile function can be improved by NBD treatment, but given its ability to dampen both the inflammatory response and stimulate new skeletal muscle growth resulting in improved contractile function, testing the potential of NBD to improve cardiac function in a DMD-related model of cardiomyopathy is warranted. To this end, we focused our current investigation on translating the basic finding of effective NF-κB inhibition into improved cardiac contractile function. We used a model of DMD that is known to have a more severe cardiac dysfunction than the *mdx *mouse. In this double knock-out (dko) mouse, where both dystrophin and its partially compensating homolog utrophin are both absent[9], we previously showed that cardiac contractile function at 8 weeks-of-age[10] is severely affected. These relatively young dko mice[10] display the classic pathophysiological hallmarks of end-stage human cardiac failure with a reduced contractile ability, a negative force-frequency relationship[11], and a severely blunted β-adrenergic response[12]. In addition these dko mice show cardiac muscle degeneration and by 10 weeks of age they have replacement of damaged cardiomyocytes with fibrotic scars[13], similar to both DMD patients [14] and the larger heart failure population[15,16]. Therefore, improvement in cardiac function in these mice would have possible therapeutic implications not only for cardiomyopathy in the muscular dystrophies, but also possibly for the much larger population of heart failure patients suffering from cardiac contractile dysfunction.

In this study, to completely assess functional aspects of NBD treatment, we investigated both the baseline contractile function of the myocardium and the regulation of contractility in the dko mice. We assessed length-dependent activation, frequency-dependent activation, and β-adrenergic stimulation in isolated dko cardiac papillary muscles treated with NBD peptide or vehicle. The results indicate that NBD can significantly improve cardiac contractile dysfunction in this model of muscular dystrophy cardiomyopathy.

## Methods

### Mice

Utrophin/dystrophin-deficient double knockout (*utrn*^*-/-*^*;mdx*, dko) offspring were born at an approximately 1:4 ratio from matings between *utrn*^*+/-*^*;mdx *mice. Offspring were genotyped shortly after birth as described previously[9] and both male and female dko mice were used for treatment and control groups. Experimental protocols involving mice were approved by the Institutional Animal Care and Use Committee at The Ohio State University.

### Peptide synthesis

Peptide synthesis of NBD was the same as described previously[5].

### Treatment regimen

Treatment with NBD was initiated when mice were less than one week of age. NBD diluted in 10% DMSO in phosphate buffered saline (PBS) was delivered by intraperitoneal injection 3 times weekly until the mice were 8 weeks-of-age. Because mice were actively growing during the first half of the treatment time and as adults their weights are variable, dko mice were weighed prior to each injection until 4 weeks of age and then once each week thereafter to achieve the desired 10 mg/kg peptide dosage. In previous studies scrambled peptide sequences showed no functional differences versus vehicle alone[5,17]. The control group for this study consisted of dko mice that were injected on the same schedule with an equal volume of the vehicle (10% DMSO in PBS).

### EMSA and Western Blotting

EMSA and western analyses were performed as previously described for skeletal muscle tissue [5,6,18] from cardiac ventricular tissue from vehicle or NBD treated dko mice. Heart tissue was homogenized and cytoplasmic extracts were prepared using an extraction buffer with standard protease inhibitors. After incubation and mild centrifugation, nuclear extracts were further isolated by using two pellet volumes of extraction buffer and standard protease inhibitors. Nuclear pellets were resuspended by vortexing and transferred to fresh tubes for use in EMSA analysis. These prepared nuclear extracts were either incubated with a radioactive oligonucleotide containing a consensus NF-∣B binding site and fractionated on a 5% non-denaturing polyacrylamide gel (EMSA) or used in a western blot and probed against p65.

### Assessment of contractile physiology

At the end of the treatment regimen, contractile function of cardiac muscle tissue was assessed *in vitro*, as previously described[10,19,20]. Briefly, under deep anesthesia, hearts were rapidly removed, and flushed with a Krebs-Henseleit solution. The right ventricle was opened, and small papillary muscles were dissected under a stereo microscope. The muscles were mounted in an experimental chamber, superfused with Krebs-Henseleit solution, containing 1.5 mM Ca^2+^, at 37°C. Muscles were electrically stimulated to twitch contract, and force of contraction was recorded. First, after the muscle had equilibrated in the set-up, muscle length was increased until a further increase in length no longer resulted in an increase in active twitch developed peak force. This length was then considered optimal length. Because the heart regulates contractile force through several physiological mechanisms, it is important not only to assess baseline contractile parameters, but also the response to normal physiological regulatory mechanisms. Therefore, we assessed the main three mechanisms used by the heart to regulate contractile strength: length-dependent behavior, frequency-dependent stimulation, and β-adrenergic stimulation. After assessment of baseline contractile parameters, at a stimulation frequency of 4 Hz, these three regulatory responses were assessed in each muscle, using protocols described previously[10,19]. The experimenters were blinded to the treatment of the mice. If more than 1 muscle was measured per mouse, data were averaged to reduce variability. N-numbers reported reflect numbers of mice studied.

### Histology

After cardiac muscle samples for physiological analyses were removed, the remaining heart tissue was frozen in Optimal Cutting Temperature (O.C.T.) medium (Tissue-Tek, Torrance, CA) on liquid nitrogen-cooled isopentane. Serial cryosections (8 μm) were cut from the tissue blocks and used for the following staining procedures. For viewing of gross histology, sections were fixed in 100% ethanol and then stained with hematoxylin and eosin using standard procedures. For specific detection of fibrosis, fibroblasts, and immune cells in regions of cardiac damage, immunofluorescence was performed on serial cryosections. Unfixed cryosections were equilibrated in KPBS (16.4 mM K_2_HPO_4_, 3.6 mM KH_2_PO_4_, 160 mM NaCl) for 5 minutes then blocked with KPBS + 1% gelatin for 15 minutes. Slides were washed with KPBS + 0.2% gelatin (KPBSG), then incubated for two hours with primary antibodies, which were diluted in KPBSG + 1% normal goat serum, against collagen I (Abcam, Cambridge, MA, ab292 rabbit polyclonal) at 1:200, ER-TR7 (Abcam ab51824 rat monoclonal) at 1:100, or CD45 (BD Pharmingen, Franklin Lakes, NJ, 550539 rat monoclonal) at 1:50. Slides were washed and then incubated for one hour with Cy3-conjugated goat secondary antibodies against rabbit IgG (Jackson Immuno Research, West Grove, PA,111-165-144) or rat IgG (Jackson Immuno Research 712-165-153), diluted 1:100 in KPBSG + 1% normal goat serum, for detection of bound primary antibodies. Slides were again washed, and then mounted in Vectashield (Vector Labs, Burlingame, CA) containing 2 μg/ml DAPI (Sigma, Saint Louis, MO) to stain nuclei. Fluorescence was viewed with a Nikon Eclipse 800 microscope (Nikon Corporation, Tokyo, Japan) and imaged with a SPOT-RTslider digital camera and SPOT software (Diagnostic Instruments, Inc., Sterling Heights, MI). Control experiments using secondary antibodies only revealed no staining.

### Statistics

Contractile forces were analyzed using unpaired t-tests or ANOVA, followed by post-hoc tests where applicable. A two-tailed P value of < 0.05 was considered significant.

## Results

**Figure 1:**
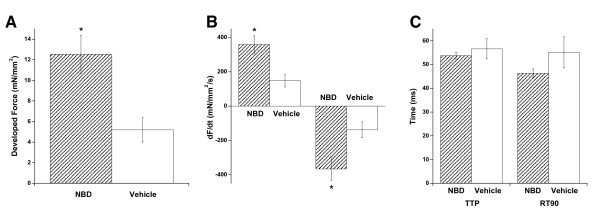
**Baseline contractile function**. **A**. Muscles from NBD treated dko mice (n = 9 muscles from n = 7 mice) exhibited a higher active developed force under baseline conditions (1.5 mM Ca^2+^, 4 Hz, 37°C) compared to muscles from vehicle treated control dko mice (n = 5 muscles from n = 4 mice). **B**. Maximum and minimum derivative of force (dF/dt) was higher in NBD treated mice. **C**. Time from stimulation to peak tension and time from peak tension to 90% relaxation were slightly, but not significantly, slower in non-treated muscles. * indicates a difference of P < 0.05 between the two groups.

At 8 weeks-of-age after treatments three times per week (starting in the first week of life) with NBD peptide (NBD) or an equivalent volume of vehicle, functional and histological parameters of dko hearts were assessed. Contractile strength of isolated multicellular cardiac muscles was first examined. These linear muscle preparations contain cardiomyocytes, fibroblasts, and endothelial cells, and are arranged in a linear fashion facilitating both qualitative and quantitative assessment of mechanical function and its regulatory process[21,22]. At baseline conditions (optimal length, 4 Hz stimulation frequency, 37°C), active force development in muscles from NBD treated dko mice was significantly higher than in muscle from vehicle treated dko mice (12.5 ± 1.8 vs. 5.2 ± 1.8 mN/mm^2^, P < 0.05, Figure 1A). Quantitatively, this difference is similar to that observed between healthy wild type (WT) mice and dko mice in our previous study[10], indicating a full recovery of active developed force by NBD. The diastolic tension needed to reach optimal active tension was not significantly different between the two groups, and was 11.7 ± 1.9 mN/mm^2 ^in the vehicle group, and 10.8 ± 1.9 mN/mm^2 ^in the NBD treated group (P = 0.75). The maximal speed of contraction and relaxation (dF/dt_max _and dF/dt_min _respectively) was also significantly higher in muscles from NBD treated mice (P < 0.05, Figure 1B). However, the increase in the derivative of force is mainly a result from the overall increase in force. When we assessed the time from stimulation to peak tension, and the time from peak tension to 90% relaxation, we only observed a small, non-significant acceleration of contractile kinetics (Figure 1C). This too indicates an improvement in function, as often increase force development per se leads to a slowing of the relaxation[23], possibly impairing diastolic function. Clearly, despite the increased force in muscles from NBD treated mice, these relaxation kinetics were not slower, and even trended to be faster.

**Figure 2:**
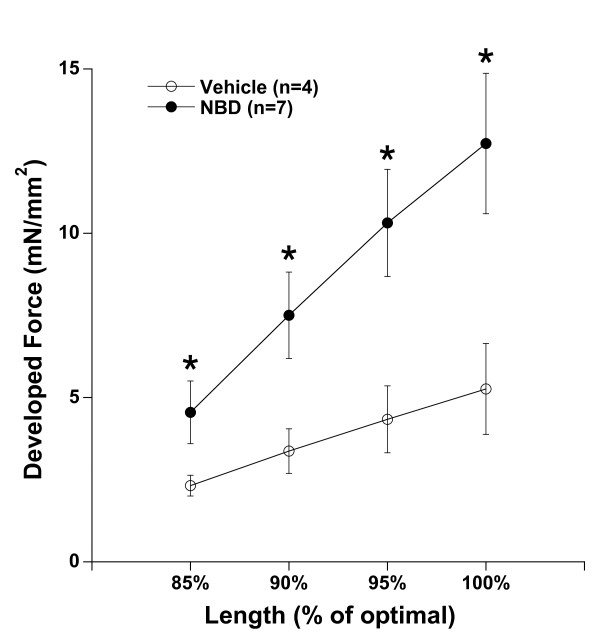
**Length-dependent activation**. When the muscle was stretched from 85% of optimal length (near slack, virtually no passive tension, 37°C) to optimal length, active force development significantly increased in both NBD treated and vehicle treated groups. Repeated measures ANOVA indicated that impact of both factors, treatment and length, were significant (P < 0.05), but not the interaction, indicating unchanged length-dependent behavior after NBD treatment in dko mice. * indicates a difference of P < 0.05 between the two groups.

In order to assess whether force development was increased independent of its regulatory mechanisms, we next investigated whether the normal physiological regulatory mechanisms that augment cardiac contractility were altered by NBD treatment. Normal physiological regulation of contractile function occurs via several mechanisms, and is used to increase blood flow when bodily demand is higher, such as occurs when exercising. The most well known of these regulatory mechanisms is the Frank-Starling mechanism, which results in an increase in contractile strength when preload (ventricular volume at start of contraction) of the ventricle, or length of the cardiac muscle cells, is increased. To mimic this mechanism in our *in vitro *preparation, we assessed contractile strength at 4 different muscle lengths (representing different loading conditions of the ventricle), ranging from 85% of optimal length, which is near-slack length of the muscle, to optimal length. We observed that length-dependent activation *per se *(shape of the curve) was not different in muscles from NBD treated compared to vehicle treated dko mice (Figure 2). Therefore, as length of the muscle increased, force of contraction increased in both groups. Statistical analysis via ANOVA indicated that the treatment difference on force was significant, as was the effect of length, but not the interaction between these two, indicating length-dependent behavior is unchanged after NBD treatment.

**Figure 3:**
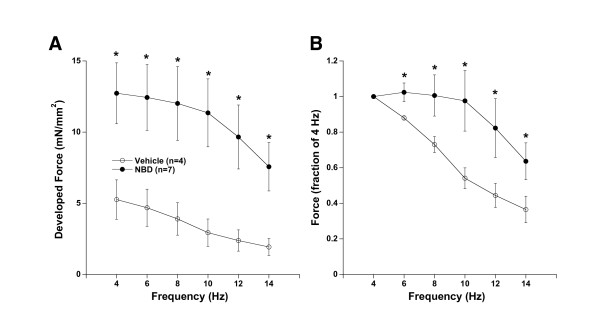
**Frequency-dependent activation**. **A**. An increase in frequency led to a decrease in force development in both muscles from NBD treated and vehicle treated dko mice. **B**. When normalized to their individual initial forces at 4 Hz, NBD treated muscles do not exhibit the negative force-frequency behavior displayed by the vehicle treated group at the lower frequency range at 37°C. All muscles were kept at their optimal length during this protocol. ANOVA (repeated measures) indicated that both the factors treatment and frequency, as well as the interaction between these two factors was significantly different. * indicates a difference of P < 0.05 between the two groups.

Next, we investigated the effect of NBD treatment on a second mechanism of cardiac contractile regulation: frequency-dependent behavior. From baseline conditions at optimal length, stimulation frequency was increased from 4 to 6, 8, 10, 12 and 14 Hz, encompassing the *in vivo *range for the mouse[24]. As we have previously shown in untreated dko mice[10], vehicle treated dko mice show a pathological negative force-frequency with an increase in stimulation rate leading to a decrease in peak contractile force. ANOVA indicated not only that both frequency and treatment were significant, but also that the interaction was significantly different between NBD and vehicle treated dko mice. Due to the spread in the absolute forces, this cannot be easily illustrated from the absolute force values (Figure 3A) but when each muscle is normalized to its own initial force level at 4 Hz, this relationship is more easily represented (Figure 3B). In vehicle treated mice a shift from 4 to 10 Hz stimulation frequency resulted in a 46 ± 6% loss of force (p < 0.05, negative force-frequency). In contrast, in NBD-treated mice the change in force from 4 to 10 Hz stimulation frequency was not significant. This flat force-frequency relationship is again nearly identical in quality and quantity compared to results obtained in healthy WT mice[10]. Thus, NBD treatment significantly prevented a worsening of frequency-dependent behavior of the muscles. When stimulation rate increased, both groups responded with a virtually equal increase in the rate of kinetics. The average acceleration of the 50% relaxation time was 10.2 ms in NBD treated mice versus 9.8 ms in vehicle treated dko mice (not shown, difference not significant).

**Figure 4:**
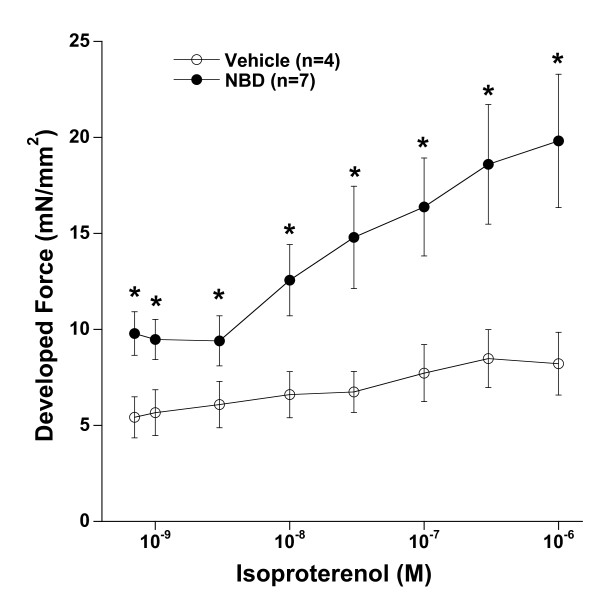
**β-adrenergic response**. The severely blunted response to the β-adrenergic agonist isoproterenol in muscles from dko mice is significantly ameliorated by NBD treatment. ANOVA (repeated measures) indicated that both the factors isoproterenol and frequency, as well as the interaction between these two factors was significantly different between NBD and vehicle treated groups. Stimulation frequency was 4 Hz, at 37°C. * indicates a difference of P < 0.05 between the two groups.

The third major mechanism that regulates contractile function *in vivo *is β-adrenergic stimulation. In order to assess this response, we exposed the twitch contracting muscles to increasing concentrations of the β-adrenergic agonist isoproterenol. As shown in Figure 4, the response in vehicle treated dko muscles to isoproterenol is pathologically weak, with an average increase in force of only 2.8 mN/mm^2^. This weak response is in close agreement with our previously published findings[10]. In sharp contrast, the response in NBD treated dko mice is robust, more than triple (average of 10.0 mN/mm^2^) than the response observed in vehicle treated mice. Again, this restored response was similar in magnitude to that of healthy wild-type mice in our previous study[10]. The acceleration of relaxation was similar in both groups, and not significantly different (not shown).

**Figure 5:**
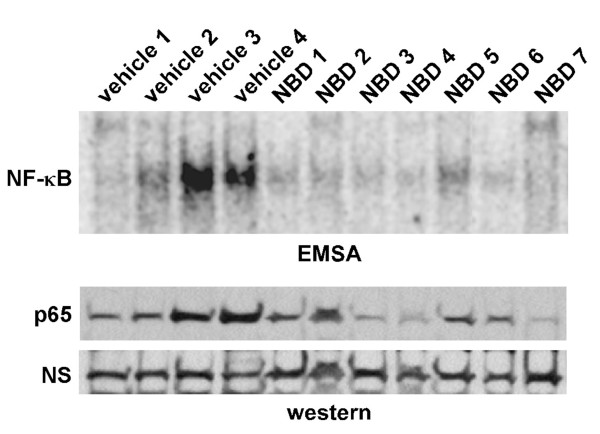
**NBD is effective in inhibiting NF-κB in cardiac muscles from dko mice**. Nuclear extracts were prepared from hearts of vehicle (n = 4) or NBD (n = 7) treated dko mice and analyzed by either EMSA (upper panel) or western blot probing for nuclear fraction p65 (bottom panels). Nonspecific band (NS) is shown on the western blot to demonstrate equivalent protein loading.

Next, we examined the pharmacodynamic efficacy of the NBD peptide in cardiac muscles of dko treated mice. Both NF-κB DNA binding activity, as well as nuclear levels of the p65 subunit of NF-κB were elevated in the dko heart. In general, this activation was effectively reduced in NBD treated dko mice (Figure 5). These results were consistent with our previous findings in diaphragm muscles from NBD treated mdx mice[5,6], together supporting that NBD improvement of cardiac contractile dysfunction in dko mice occurs through the inhibition of the NF-κB signaling pathway.

Lastly, we investigated whether NBD treatment of dko mice resulted in an improvement in cardiac histopathological features of this model. Between eight and ten weeks-of-age, dko mice display myocardial damage followed by fibrotic scarring in damaged regions[13]. Despite the robust improvement in contractile function resulting from NBD-treatment, and the well-documented role of NF-κB in inflammation, histopathological features of the dko myocardium were not markedly improved by NBD treatment.

**Figure 6:**
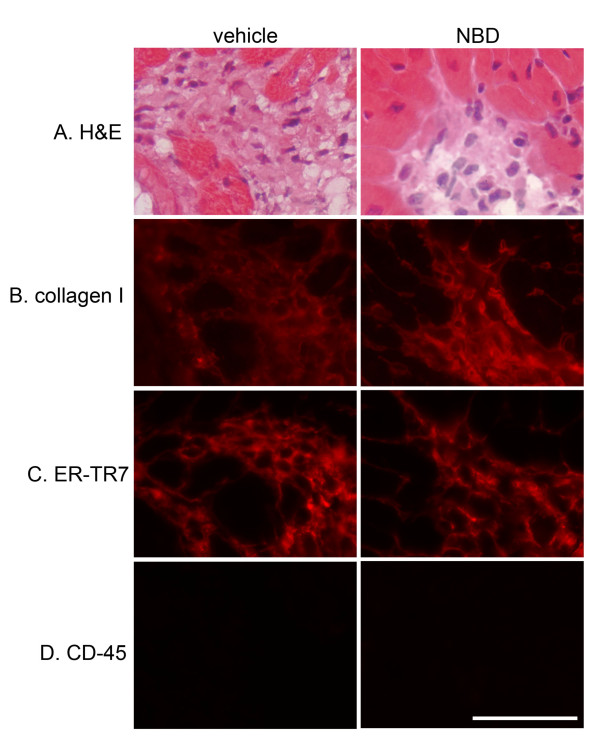
**Histological analyses of tissue damage indicators in representative serial sections of hearts from vehicle and NBD peptide treated dko mice show similar pathology in both treatment groups**. **A**. Hematoxylin and eosin (H&E) staining shows the presence of fibrotic scars in dko hearts from vehicle and NBD-treated groups. **B**. Immunostaining for collagen I shows localization of collagen in fibrotic regions. **C**. ER-TR7 immunostaining demonstrates fibroblasts are a major cellular infiltrate in regions of fibrosis. **D**. CD-45 immunostaining shows that immune cells are not detected in fibrotic scars at the time-point of analysis. Scale bar equals 50 μm.

We observed large fibrotic scars (Figure 6A) in the hearts of most of dko mice in this study regardless of treatment (5 of 7 [71%] NBD treated mice, versus 3 of 4 [75%] vehicle treated mice). Of note, the eight week-old vehicle and NBD treated dko mice in this study that were handled for injections three times per week, showed more advanced cardiac damage than other dko mice analyzed at eight weeks-of-age over the past decade that underwent minimal handling (data not shown). The amount of damage in both groups of dko mice in this study was more consistent with the damage present in ten week-old dko mice [13]. Immunofluorescence using collagen I antibodies showed that the fibrotic regions were highly collagenous in both groups (Figure 6B). Fibroblasts, known to be responsible for much of the cardiac remodeling in cardiomyopathy via secretion of matrix metalloproteinases and collagen[15], are present in large numbers in both NBD and vehicle treated dko hearts in regions of fibrosis (Figure 6C). Immune cell infiltrates are likely required for clearing damaged myocardial tissue, but at the time-point analyzed here, we could not detect the presence of more than a very few sporadic hematopoietic-lineage cells in damaged regions of hearts from either NBD or vehicle treatment groups using antibodies that recognize the general hematopoietic markers CD-45 (Figure 6D) or CD-11b or the more specific macrophage marker F4/80 (data not shown). Intermediate timepoints to quantifiably assess the inflammatory response were beyond the scope of this end-point driven study.

## Discussion

Cardiac contractile dysfunction is one of the leading causes of death in DMD. Clinical treatment of this debilitating aspect of DMD is paramount in extending both life-span and quality of life. In this study we showed that a peptide referred to as NBD which blunts NF-κB signaling, can restore cardiac contractile dysfunction in a mouse model of DMD. Not only did NBD treatment increase contractile force substantially, it also improved key governing mechanisms of contractile force that are typically impaired in patients with heart failure including force-frequency behavior and the response to β-adrenergic stimulation[11,12,25].

**Figure 7:**
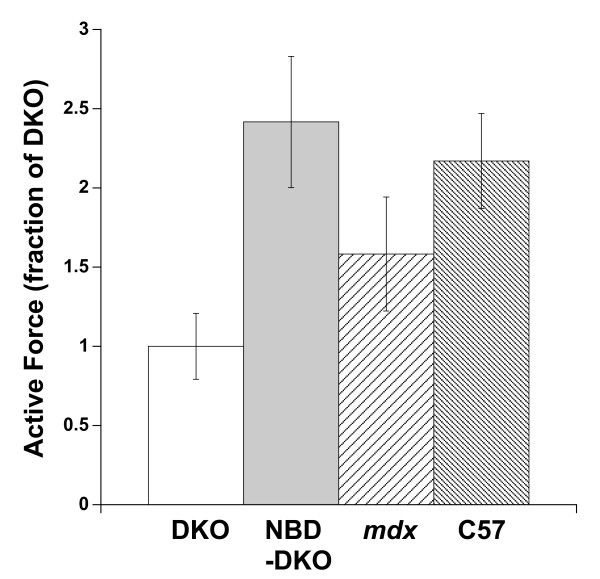
**Indirect comparison of functional improvement of dko myocardium by NBD treatment shows that the functional improvement in baseline cardiac contractile force (4 Hz, optimal length, 37°C) resulted in forces that are comparable with age-matched C57BL/10 wild type muscles, and relatively exceed those assessed in *mdx *myocardium under identical experimental conditions**. Data from this study and from Ref. [10].

For this proof-of-principle study, we did not include additional models of muscular dystrophy or wild-type mice. However, we can compare the contractile response to our previous study[10] in which we used healthy, wild-type mice as well as *mdx *(dystrophin deficient) mice. *Mdx *mice are the genotypic, often-used model of DMD with a much milder phenotype (less contractile dysfunction) compared to dko mice. In our current study, we used the small right ventricular papillary muscle with an average muscle dimension of 266 ± 8 μm wide, 177 ± 5 μm thick in the center, and 1.04 ± 0.08 mm long. In our previous work[10], we used right ventricular thin trabeculae from *mdx*, dko mice, and C57Bl/10 isogenic controls. The trabeculae used previously were slightly narrower (average width of 220 μm) and longer (average 1.5 mm). However, although trabeculae are very well suited for assessment of contractile function in general[26], their frequency of occurrence is less predictable than the always-present papillary muscles. In this study we chose to use papillary muscles based on their frequency of occurrence (i.e. increased success rate of experiment) together with the short life-span of the dko mouse (~10-12 weeks). When we normalize both studies to the dko mouse contractile force, shown in Figure 7, we can deduce that the improvement in contractile force is very substantial. In fact, forces produced during baseline conditions in NBD treated dko mice are relatively similar to those obtained in C57 wild-type mice, and higher than those obtained in untreated *mdx *mice. In addition, the responses to increased stimulation frequency as well as to β-adrenergic stimulation in NBD treated dko mice closely mimic those observed in healthy C57 wild-type mice[10].

The increased contractile strength was likely not a direct effect of altered histology of the myocardium. We observed no significant reduction in fibrosis in the dko myocardium upon treatment with NBD. However, we cannot at this point exclude that local improvements in the histology of papillary muscles may play a role. Most of the area of the right ventricle and septum where the muscles were excised is unsuitable for histological analysis due to the dissection. The muscles used for physiological force measurements, after experimentation, are also not suitable for histological analysis and subsequent correlative analysis. Thus, we cannot show a potential histological change in the preparations where function was actually assessed. However, given the widespread fibrosis that was still clearly present in the remaining ventricular tissue after NBD treatment, a local improvement of histopathology being primarily responsible for the improved function is quite unlikely. At present, and well beyond the scope of this proof-of-principle study, we can only speculate about the underlying molecular events that ultimately result in an improvement of contractile function.

The underlying cause of weakened contractile performance of the end-stage heart failing myocardium is often independent of the originating cause of heart failure in a patient or animal model. Impaired calcium handling is a central finding in end-stage heart failure, and this impaired calcium handling correlates with the blunting, or even loss, of frequency-dependent activation. In human heart failure, the normal positive force frequency response is typically severely blunted, or even becomes negative, and is a hallmark of the phenotypic dysfunction[11,24,25]. In normal, healthy mice, when frequency of contraction is increased, the force development of the muscle is generally slightly increased[27] or at least does not show a major decrease, while relaxation is always faster[19]. However, in mice with cardiac dysfunction, such as the dko mouse used in this study, the force-frequency relationship is clearly negative[10]. We show that NBD treatment not only increases contractile strength of dko myocardium, but it also significantly improved the force-frequency relationship. This response was no longer largely negative, and even reverted to positive at the lower end of the frequency range, resembling the frequency-dependent behavior typically found in healthy mice. The restoration of a normal force-frequency response is thus indirect evidence that calcium handling improvement may be the major underlying factor in the functional improvement of dko myocardium after NBD treatment.

NF-κB and calcium ions are both multifaceted signaling molecules and interactions between calcium ion concentration and NF-κB have been documented. For instance, in smooth muscle, NF-κB is negatively impacted by calcium channels[28], and thus inhibition by NBD could potentially upregulate these calcium channels, improving function by facilitating calcium influx. Also, inhibition of NF-κB has been shown to be able to alleviate sarcoplasmic reticulum stress, and interact with levels of the sarcoplasmic/endoplasmic reticulum calcium ATPase (SERCA), which is responsible for the uptake of calcium ions from the cytoplasm[29]. NF-κB in skeletal muscle has been shown to modulate expression of nitric oxide synthase (NOS) isoforms[30], which play an important role in maintaining cardiovascular homeostasis mainly via calcium handling. Lastly, a recent report by Panama and colleagues [31] showed that NF-κB downregulates the transient outward potassium current in the heart, further providing evidence for a role of NF-κB regulated processes in excitation-contraction (EC)-coupling. Therefore, although we have no direct conclusive evidence at this stage, NBD may improve contractile function in dko myocardium via improvement in EC-coupling/calcium handling, rather than via a prevention of cardiac histologically-detectable damage. Dystrophic skeletal muscle function can be improved by low levels of dystrophin in absence of histopathological improvement[32]. Therefore, a similar improvement of function of non-fibrotic dystrophic myocardium may account for the results of our study. Further targeted studies are required to elucidate possible mechanisms and could include electrophysiological and heamodynamic assessments[33,34], as well as intracellular calcium handling[19]. Any therapeutic strategy involving NBD may require a combinatorial approach with a factor that would prevent cardiac damage

In addition to reduced contractility and a negative force-frequency response, it is well known that both in patients with heart failure, as well as in many animal models of cardiac dysfunction, the physiological response to β-adrenergic stimulation is severely blunted[12]. In untreated dko myocardium, this blunted β-adrenergic response is typically observed, and is severe[10], and in the present study we found that NBD treatment significantly improves this response. The main underlying molecular level events that lead to increased contractility after β-adrenergic stimulation may again be found in the enhancement of the intracellular calcium transient. Thus, the same mechanism responsible for the improved force-frequency response could be the main factor for improvement of this β-response.

## Conclusions

In this study we show that inhibition of NF-κB using the small peptide inhibitor NBD improves contractile force, improves the force-frequency relationship, and restores the response to β-adrenergic stimulation in the well-established murine model for cardiac dysfunction associated with DMD. Since we have demonstrated a therapeutic effect of NBD on both skeletal[6] and cardiac muscle (this study), NBD peptide treatment may be a realistic treatment option for this debilitating disease. Moreover, because the dko mouse model recapitulates many of the contractile phenotypes found in the majority of patients with end-stage failure stemming from a variety of etiologies, NBD treatment may be useful beyond the field of muscular dystrophy.

## Competing interests

The authors declare that they have no competing interests.

## Authors' contributions

DAD performed the histology, bred and genotyped the dko mice. YX performed the muscle experiments and analyzed the data. JP designed the treatment regimen, treated the mice, and performed EMSA experiments. PMLJ, DG, and JRF designed the study, PMLJ performed data analysis and statistics, and wrote the initial manuscript. JRF verified histological data, and DCG and JRF wrote specific sections, reviewed, and edited the whole manuscript. All authors read and approved the final manuscript.
